# A clinical decision support system improves antibiotic therapy for upper urinary tract infection in a randomized single-blinded study

**DOI:** 10.1186/s12913-020-5045-6

**Published:** 2020-03-06

**Authors:** M. Neugebauer, M. Ebert, R. Vogelmann

**Affiliations:** Second Department of Internal Medicine, University Medical Center Mannheim, Medical Faculty Mannheim, Heidelberg University, Theodor-Kutzer-Ufer 1-3, D-68167 Mannheim, Germany

**Keywords:** Clinical decision support system, Upper urinary tract infection, Empiric antibiotic therapy, Antibiotic resistance - UTI

## Abstract

**Background:**

Due to increasing bacterial resistance rates choosing a correct empiric antibiotic therapy is getting more and more complex. Often medical doctors use information tools to make the right treatment choice.

**Methods:**

One hundred sixty six participants (77 medical doctors and 89 medical students) were asked to provide a diagnosis and antibiotic therapy in a simple fictive paper case of upper urinary tract infection (UTI) in a randomized single-blinded study. Participants were randomized to one of four information tools they were allowed to use in the study or control: 1. free internet access, 2. pharmaceutical pocket guide, 3. pocket guide antibiotic therapy, 4. clinical decision support system (CDSS), and control (no information tool). The CDSS was designed for the study. The adherence to the national German UTI guideline was evaluated.

**Results:**

Only 27.1% (*n* = 45/166) provided a correct diagnosis of upper UTI and 19.4% (*n* = 32/166) an antibiotic treatment recommended by national German treatment guidelines indicating their need for information tools. This result was not significantly different between medical doctors and medical students, residents and medical specialists or level of working experience. Using CDSS improved results significantly compared to conventional tools (diagnosis 57.1%; treatment recommendation 40.5%; *p* < 0,01). Processing time was not different between the use of CDSS and conventional information tools. CDSS users based their decision making on their assigned information tool more than users of conventional tools (73.8% vs. 48.0%; *p* < 0.01). Using CDSS improved the confidence of participants in their recommendation significantly compared to conventional tools (*p* < 0.01).

**Conclusions:**

Our study suggests that medical professionals require information tools in diagnosing and treating a simple case of upper UTI correctly. CDSS appears to be superior to conventional tools as an information source.

## Background

Antibiotic prescriptions play a central part in modern medicine as causative therapy for infectious diseases [1, 2]. Due to irrational prescribing practices, the rate of multiresistant bacteria has dramatically increased over the last years, and it is more and more challenging to recommend effective empiric antibiotic therapy [3, 4]. Despite these circumstances, medical doctors often continue to prescribe antibiotics unreasonably in their daily routine [5, 6].

### Clinical decision support system

Antibiotic Stewardship (ABS) Programs are implemented to ensure the rational use of antibiotics [7]. These programs aim to optimize antibiotic prescriptions hence to increase guideline adherence, often leading to a reduction in overall antibiotic use [7]. Because of their effectiveness, ABS programs have become an integral part of medicine in the last years [8, 9].

ABS programs recommend the use of Clinical Decision Support Systems (CDSS) to improve antibiotic therapies [7]. CDSS are software programs supporting medical doctors in their decision-making process. They can integrate large databases, e.g., local resistance data, pharmaceutical data, or medical guidelines, and highlight important facts for various treatment options. Available CDSS systems for antibiotic therapy are often active interventions that give specific recommendations [8]. In theory, CDSS has the potential to increase therapy safety and guideline adherence, but current studies reveal mixed results about their efficacy [9, 10].

In the digital age, computer programs become more and more important in hospitals and outpatient office [11]. Medical students and doctors use smartphone apps more frequently [12]. These apps can improve antibiotic therapy concerning local antibiotic resistance [13]. Therefore, CDSS will play an essential part in ABS programs in the future and could eventually replace current information tools like textbooks or pocket guides.

### Urinary tract infection

Urinary tract infection (UTI) is a common infectious disease for in- and outpatients worldwide.

[14]. Treatment guidelines distinguish between a lower and upper urinary tract infection, as this determines the choice of antibiotic treatment [15, 16]. Urinary tract infection is defined as symptoms pertaining to the urinary tract such as dysuria, pollakiuria, and/or imperative urgency. In upper urinary tract infection, additional symptoms such as renal angle tenderness, flank pain and/or fever (> 38 °C) are present. Due to bacterial resistance, it is challenging to recommend effective empiric antibiotic therapy [17]. Up to date, only one study analyzed the effect of a CDSS on compliance with guidelines on antibiotics prescribed for urinary tract infection [18]. In this study, the CDSS was used at the point of care on desktop computers in the emergency department in three study centers. When a urinary tract infection diagnosis was validated, the CDSS was automatically triggered at the computer. The system collected data automatically from the electronic medical record, and the user could add additional data. The initial diagnosis could be revised, and it displayed recommendations regarding further diagnostics, indications for hospitalization, antibiotic treatment, and follow-up based on national urinary tract infection guidelines. The clinician could decline to use the CDSS [18].

We developed a CDSS for the current study as an active intervention with a system that gives recommendations to diagnosis and therapy for urinary tract infections based on national German urinary tract infection guidelines. Similar to the CDSS by Demonchy et al., participants could decline to use the CDSS, individual patient data could be added, and our system recommended the diagnosis, antibiotic treatment including dosing and duration tailored to individual patient data [18]. In contrast, participants in our study were presented with a simple fictive case of upper urinary tract infection. The hypothesis tested is that a CDSS with specific recommendations based on individual patient data and national treatment guidelines significantly improves adherence to the national German urinary tract infection guideline compared to conventional information tools or no intervention.

## Methods

### Participants and study settings

The target group was defined as medical doctors with completed medical training qualified to practice medicine in Germany independent from their specialty. Besides, medical students in their clinical stage of medical training (year 3–6 in German medical school) were asked to participate as well. An invitation was sent by Email to medical doctors identified by the mailing list of the 2nd department of Internal Medicine at the University Medical Center Mannheim and students via the mailing list of the student association, medical faculty Mannheim, University Heidelberg. Information flyers were distributed at the medical library, medical faculty Mannheim, University Heidelberg. Detailed information about time and location for participation at the University Medical Center Mannheim was provided on the website http://www.vera-studie.de, which was shared by the social media platform ‘Facebook’. Potential participants were also directly addressed at the Annual Meeting of the German Society for Digestive and Metabolic Diseases (DGVS) 2014 in Leipzig, the German Society for Internal Medicine (DGIM) 2015 in Mannheim and at the University Medical Center Mannheim. The study was conducted at the convention centers of the DGVS 2014 meeting in Leipzig (Sept 18–20, 2014), the DGIM 2015 meeting in Mannheim (April 18–19, 2015), or scheduled appointments at the University Medical Center Mannheim.

### Study design and intervention

The study design was a single-blinded, randomized controlled study. After completing a form for data protection and providing personal information (Fig. S1), participants were randomized by using the random generator https://www.zufallsgenerator.net for the numbers 1–5: 1. Free Internet Access (FIA); 2. Pharmaceutical Pocket Guide (PPG) [19]; 3. Pocket Guide Antibiotic Therapy (PGAT) [20]; 4. CDSS and 5. control (no information tool). The participants had to identify themselves with their name, surname, and date of birth on the form for data protection, and the results of the study where then anonymized using a participant’s ID. This method allowed us to confirm that participants did not repeat the test. A case of uncomplicated upper urinary tract infection was provided, and participants were asked to name diagnosis and to recommend an empiric antibiotic therapy (Fig. S4). The participants were pointed to the fact that only complete result sheets with diagnosis and antibiotic therapy were eligible for the study. They were allowed to use the assigned information tool in order to identify the correct diagnosis and empiric therapy based on German national guideline recommendations [15]. Neither the guideline information nor a summary of the evidence to support the recommendation was given to the participants. We asked the participants if they used the assigned information tool for answering the questions. Only participants using the free internet access were asked, which websites they used. For all other media no data logs or interviews were performed to analyze their search strategy. The time to work on the case was taken from handing out the paper case to its return. Afterward, participants were asked to provide details about their decision making and the assigned information tool (Fig. S2).

### Clinical decision support system

A CDSS was newly designed for the study. It is a server-based software named “Infektionsnetzwerk/Antibiotix”, which was accessible via an internet web browser. The software integrated the German national guidelines for uncomplicated urinary tract infection [15] and a commercial pharmaceutical database ‘ifap systemDATEN’ (ifap GmbH; Munich, Germany). A search field allowed to enter the assumed diagnosis (Fig. S3A) and based on disease characteristics or symptoms suggested by the CDSS (Fig. S3B) the software tool provided a list of potential diagnosis ordered by likelihood (Fig. S3C). Next, the user was pointed to a recommended empiric antibiotic therapy for the diagnosis of choice. Additional parameters like glomerular filtration rate (GFR), weight, size, and current co-medication could have been entered, and the system recommended dose adjustments or hinted to potential drug interactions.

### Data collection and outcomes

Answers were graded by a blinded reviewer based on current German national guidelines for uncomplicated urinary tract infection independent of the assigned information tool [16]. The result sheet did not contain any information about the information tool. The primary outcome was defined as adherence to the guidelines in diagnosis and treatment. Upper urinary tract infection, pyelonephritis, ascending urinary tract infection, or urosepsis was defined as correct diagnosis. Ciprofloxacin and levofloxacin were considered as the recommended first-line therapy and cefpodoximproxetil and ceftibuten as second-line. Because of a slight decrease in renal function, the dose needed to be adjusted according to official drug information. A treatment choice was considered incorrect if an antibiotic other than the recommended first- or second-line therapy was named, the length of therapy or the dose adjustment was incorrectly stated. The secondary outcome was processing time and confidence in diagnosis and treatment choice. Only questionnaires with provided information for the current workplace, diagnosis, and therapy were analyzed. Questionnaires with incomplete information were included for subgroup analysis if reasonable. Therefore, the number of used questionnaires differs in subgroup analysis. We performed an Intention-to-use (ITU) and an As-used (AU) analysis based on the feedback of the participant. The ITU analysis included all participants, regardless of the actual use of the assigned information tool. The AU analysis included only participants who indicated in the second questionnaire that they used the assigned tool for completing the assignment (Fig. S2).

### Statistical analysis

Data were analyzed using the statistic software Stata/IC 13.1 for Mac (64-bit Intel) Revision 16 Dec 2016 (StataCorp, USA) and Microsoft® Excel for Mac Version 16.11.1 (180319) (Microsoft®, USA). Working time and choice of antibiotic therapy were analyzed using the Shapiro-Wilk test and the Shapiro-Francia test for normal distribution. Statistical significance was analyzed using the analysis of variances (ANOVA), the Bonferroni correction, Chi^2^-tests, and Fisher‘s exact test (*p*-value < 0.05 was defined as ‘significant’).

## Results

### Participants characteristics

One hundred seventy six medical doctors and students participated in the study, 7 participants did not fill out the paperwork correctly, and 3 did not meet requirements and were excluded. Therefore, 166 participants were included in the analysis, 46.4% (*n* = 77/166) medical doctors and 53.6% (*n* = 89/166) medical students (Table 1). The average working experience of medical doctors was 12.9 ± 9.7 years, 32% (*n* = 23/72) were practitioners in outpatient offices, and 68% (*n* = 49/72) worked in a hospital. The majority were medical specialists (67.1%; *n* = 51/76), mainly internal medicine (*n* = 38/50) with a working experience of 18.2 ± 7.6 years compared to residents (32.9%; *n* = 25/76) with a working experience of 2.7 ± 2.1 years. Only eight medical students in their last year of medical training (year 6) participated (9%; *n* = 8/89) with the majority of medical students in year 3–5 (91%; *n* = 81/89; Table 1).

**Table 1:** Sociodemographic data

Variable	Value	n
**Age (years)**	31.6 ± 11.1	165
**Gender**		165
**Male**	88 (53.3%)	165
**Female**	77 (46.7%)	165
**Level of education**		166
**Medical doctors**	77 (46.4%)	166
Working experience (years)	12.9 ± 9.7	73
**In hospital**	49 (68%)	72
**In outpatient office**	23 (32%)	72
**Medical specialists**	51 (67.1%)	76
**Internal medicine**	38 (76%)	50
**Surgery**	4 (8%)	50
**Pediatrics**	2 (4%)	50
**General medicine**	2 (4%)	50
**Industrial medicine**	1 (2%)	50
**Gynecology**	1 (2%)	50
**Pharmacology**	1 (2%)	50
**ENT**	1 (2%)	50
Working experience (years)	18.2 ± 7.6	48
**Residents**	25 (32.9%)	76
Working experience (years)	2.7 ± 2.1	25
**Medical students**	89 (53.6%)	166
**Clinical year 3–5**	81 (91%)	89
**Clinical year 6 (final year)**	8 (9%)	89

### Fictive case of urinary tract infection

A fictive case of uncomplicated upper urinary tract infection (pyelonephritis) was presented (Fig. S4). A 64-year-old woman (80 kg; 168 cm) enters the emergency room because of fever (39 °C) and burning while urinating. For the last 2 days, she had problems to empty her bladder, and she used the bathroom about 8 times a day only with small urine portions, no vaginal discharge. In the emergency room, a urine dipstick is positive for leucocytes, nitrate, and negative for glucose, no renal angle tenderness, body temperature 38.7 °C. Medical history: arterial hypertension, diabetes mellitus type 2. Medication: Ramipril 5 mg q.d., Metformin 500 mg b.i.d., Calcium/Vitamin D3 q.d.. Lab work: leucocytes 14.7*10^9^/l [4.2–10.2*10^9^/l], CRP 112 mg/l [0-5 mg/l], creatinine 114,9 μmol/l [58,3–96,4 μmol/l], MDRD-GFR 41.2 ml/min/1.73m^2^, serum glucose level 7.94 mmol/l [4.1–6.1 mmol/l]. Blood and urine cultures are pending.

### Overall adherence to guidelines

The correct diagnoses upper urinary tract infection, pyelonephritis, ascending urinary tract infection, or urosepsis was named by 27.1% (*n* = 45/166) in the ITU-Analysis (Table 2). There was no significant difference between students (27.0%; *n* = 24/89) and medical doctors (27.3%; *n* = 21/77). Working experience or level of education also made no significant difference in providing an accurate diagnosis. The diagnoses lower urinary tract infection or cystitis were selected in 30.1% (*n* = 50/166) and urinary tract infection in 42.8% (*n* = 71/166). The distinction between upper and lower urinary tract infection is important as antibiotic treatment varies significantly between the two entities [16].

**Table 2:**
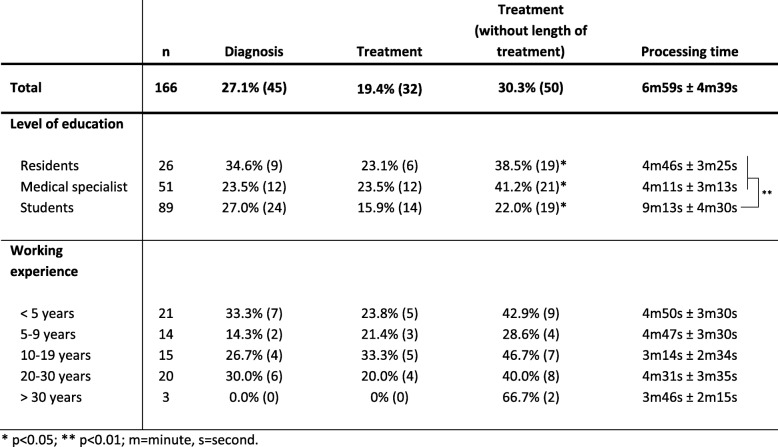
ITU-Analysis: Diagnosis, treatment, processing time

In this fictive case, the patient had a slightly increased blood sugar level in the emergency room, and the glucose level in the urine was negative, indicating a stable metabolic state hence treatment recommendations for patients with upper urinary tract infection without diabetes mellitus should be applied [16]. According to these guidelines, ciprofloxacin and levofloxacin were considered as the recommended first-line therapy and cefpodoximproxetil and ceftibuten as second-line [16]. Because of a slight decrease in renal function, the dose needed to be adjusted according to the official drug information (Table 3). A treatment choice was considered incorrect if an antibiotic other than the recommended first- or second-line therapy was named, the length of therapy or the dose adjustment was incorrectly stated.

**Table 3:** Antibiotic treatment upper UTI – modified [16]

Antibiotic	Regular dose	Dose adjustment	Frequency	Length of treatment [days]
Ciprofloxacin	500 mg – 750 mg i.v./p.o.	250 - 500 mg i.v./p.o.	b.i.d	7–10
Levofloxacin	(250 mg) – 500 mg i.v./p.o.	500 mg first dose then 250 mg i.v./p.o.	q.d.	7–10
Levofloxacin	750 mg i.v./p.o.	750 mg, first dose then 500 mg i.v./p.o.	q.d.	5
Cefpodoximproxetil	200 mg p.o.	200 mg p.o.	b.i.d.	10
Ceftibuten	400 mg p.o.	400 mg first dose then 200 mg p.o.	q.d.	10

Only 19.4% (*n* = 32/166) of the participants in the ITU-analysis suggested a recommended antibiotic treatment with dose adjustment for the patient’s GFR and with a recommended length of therapy. There was no significant difference between participants with a different level of education or working experience (Table 2). However, when the length of therapy was not considered in the ITU-analysis, overall 30.3% (*n* = 50/166) provided a correct empiric therapy, significantly more by medical doctors (residents and medical specialists) compared to medical students (*p* < 0.05). The average processing time was significantly longer in medical students compared to residents or medical specialists (*p* < 0.01; Table 2).

Participants, who did not distinguish between upper or lower urinary tract infection (42.8%; *n* = 71/166), only 11.3% (*n* = 8/71) named a recommended treatment for upper urinary tract infection with dose adjustment and correct length of treatment. Without considering the length of treatment, 28.2% (*n* = 20/71) were correct in their treatment recommendations, and 39.4% (*n* = 28/71) picked at least an antibiotic of choice according to treatment guidelines (Table 3). However, the majority of participants, who provided a more general diagnosis of urinary tract infection, named an antibiotic for treating uncomplicated cystitis (lower urinary tract infection; 60.6% (*n* = 43/71)).

### Efficacy of CDSS compared to conventional tools

The use of information tools showed mixed efficacy in diagnosis and treatment recommendations. Participants were randomized into four different groups with assigned information tools with a similar distribution of medical doctors and students among groups and control (*p* = 0.79; Fig. S5). The information tool CDSS improved the correct diagnosis significantly (57.1%; *n* = 24/42) compared to conventional information tools (19.0%; *n* = 19/100) and control (8.3%; n = 2/24; *p* < 0.01; Table 4). Free internet access compared better (29.4%; *n* = 10/34) than the use of a standard pocket guide for antibiotic treatment (17.7%; *n* = 6/34) or a pharmaceutical pocket guide (9.4%; *n* = 3/32). In the AU-analysis (participants indicated the actual use of the assigned information tool afterward), the CDSS (62.1%; *n* = 18/31) was also significantly better than conventional information tools (22.9%; *n* = 11/48), free internet access (24.1%; *n* = 7/18), pharmaceutical pocket guide (10.3%; *n* = 3/13) and pocket guide for antibiotic treatment (3.5%; *n* = 1/17) (*p* < 0.01; Table 4).

**Table 4:**
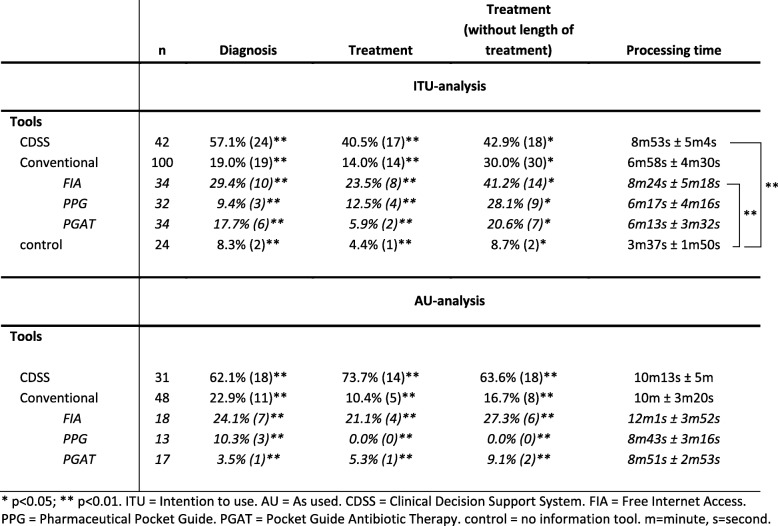
Information tools: Diagnosis, treatment, processing time

In the ITU-analysis, the efficacy in providing a guideline-recommended treatment was significantly better using CDSS (40.5%; *n* = 17/42) compared to conventional tools (14.0%; *n* = 14/100; free internet access 23.5%; pharmaceutical pocket guide 12.5%; pocket guide for antibiotic treatment 5.9%) and control (4.4%; *n* = 1/24) (Table 4). In the AU-analysis, CDSS also improved treatment recommendations (73.7%; *n* = 14/31) significantly compared to conventional tools (10.4%; *n* = 5/48; *p* < 0.01). If the length of treatment was not considered in the ITU- or AU-analysis, treatment recommendations improved with conventional information tools or control, but not with CDSS suggesting that CDSS has stronger effects on recommending the correct length of treatment (Table 4). In contrast to the length of treatment analysis, analysis without dose adjustment showed overall no significant differences compared to analysis with dose adjustment (data not shown).

### Processing time

Processing time is an essential factor in the daily routine and could affect the acceptance of an information tool. Participants using CDSS (8 min (m) 53 s (s) ± 5m4s) and free internet access (8m24s ± 5m18s) had on average the longest processing time significantly longer than control when no information tool was used (*p* < 0.01; Table 4). However, the pharmaceutical pocket guide (6m17s ± 4m16s) and pocket guide for antibiotic treatment (6m13s ± 3m32s) were not significantly faster than CDSS or free internet access.

### Use of CDSS and conventional information tools

More than half of participants based their decision making on the assigned information tool (54.1%; *n* = 79/146), and as one would expect medical students significantly more than residents or medical specialists (85.1% vs. 36.0% vs. 14.9%, respectively; *p* < 0.01). CDSS users based their decision making on their assigned information tool more than users of conventional tools (73.8% vs. 48.0%; *p* < 0.01; Table 5). Participants were asked to rate how confident they were in providing an accurate diagnosis and treatment recommendation on a scale from 1 (= very confident) to 6 (= very uncertain) afterward. They graded their recommendations on average as 2.8 ± 1.3. Residents and medical specialists assessed their recommendations significantly better than medical students (2.4 and 2.3 vs. 3.3, respectively; *p* < 0.01). Using CDSS improved the self-assessment of participants significantly compared to conventional tools, free internet access, pharmaceutical pocket guide, pocket guide for antibiotic treatment, and control (*p* < 0.01; Table 5).

**Table 5:**
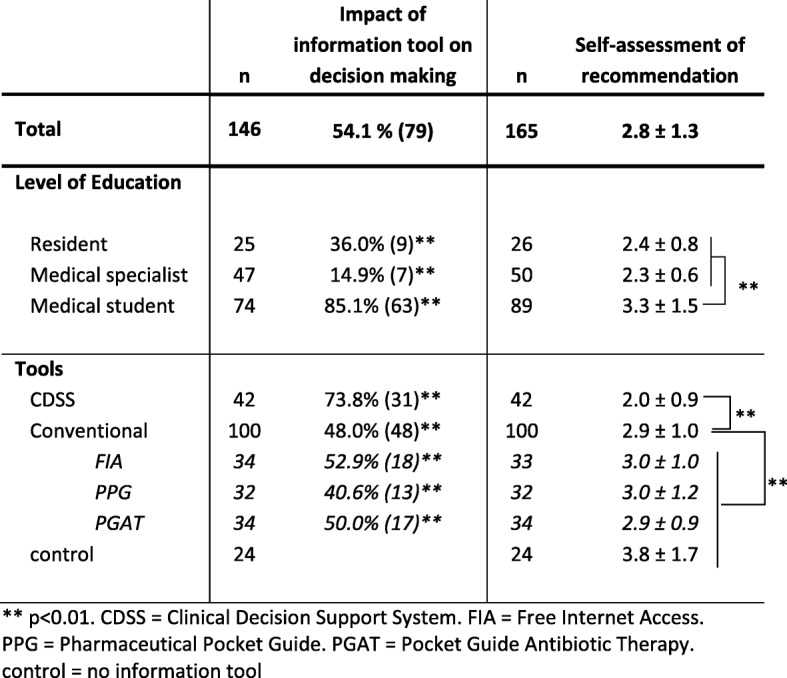
Impact of information tool on decision making and self-assessment of recommendation

## Discussion

The data presented show that the participants of this study have difficulties in adhering to the national German urinary tract infection guidelines in providing a correct diagnosis and empiric antibiotic treatment for a fictive case of upper urinary tract infection. A CDSS improves adherence to guidelines significantly compared to conventional information tools or no intervention.

### Diagnosis of upper urinary tract infection

Empiric treatment of urinary tract infection can be challenging due to increasing resistance rates of standard antibiotics [17]. However, an antibiotic treatment recommendation can only be as good as a correct diagnosis. In our study, a simple case of upper urinary tract infection was presented with symptoms pertaining to the urinary tract. All participants identified the case as a urinary tract infection, but the distinction between lower and upper urinary tract infections was weak. The majority did not distinguish between lower and upper urinary tract infections (42.8%), and besides, 30% picked lower urinary tract infection as the diagnosis. According to several international guidelines, fever > 38.0 °C points to upper urinary tract infection, and additional symptoms such as renal angle tenderness and/or flank pain are not required for diagnosing upper urinary tract infection [15, 16]. It is disturbing that only 27.1% of study participants named the correct diagnosis (upper urinary tract infection, pyelonephritis, or urosepsis) without a significant difference between medical doctors and medical students, residents and medical specialists or years of working experience. In the study of Demonchy et al., only 23% of the initial urinary tract infection diagnosis was modified after using the CDSS, indicating that the majority of diagnoses were determined correctly without using an information tool. However, determining the correct diagnosis was not analyzed and verified in this study [18]. It is unclear how many of the medical doctors in our study were experienced in treating this kind of infectious disease. However, the majority of medical doctors indicated internal medicine as their medical specialty. Urinary tract infections are one of the most common bacterial infections worldwide, so we assume that medical doctors in the field of internal medicine are acquainted with the disease.

Even participants naming the unspecific diagnosis of urinary tract infection (42.8%) picked in 60.6% an antibiotic used for lower urinary tract infection therapy, suggesting that they had an incorrect diagnosis in mind. An explanation could be a fundamental misunderstanding in interpreting the case across all participants or a lack of seriousness in working on the case. The personal interaction with a patient was missing, which could lead to a lack of feeling responsible for a real-life person. Also, we cannot exclude that participants handed on ‘false’ results to others waiting in line. However, the fictive case was constructed to be simple, representing a typical case without pitfalls, which does not require much pondering or studying of additional information, at least for trained medical doctors. Therefore, the data suggest that in our small population, medical doctors lacked knowledge of the definition of upper urinary tract infection significantly as a foundation for a proper treatment recommendation.

### Antibiotic treatment choices

Antibiotic treatment recommendations were also poor among study participants in part due to the improper distinction between upper and lower urinary tract infections. The treatment guideline for uncomplicated urinary tract infection in Germany was about 3 years old when the study was conducted [16]. A potential explanation could be that new scientific findings could have led to a deviation from national treatment guidelines in the meantime. However, the latest guidelines from 2018 had similar treatment recommendations arguing against this hypothesis [21]. National guidelines should always be adapted to local resistant patterns of bacteria, and hospitals should use local Standard Operating Procedures (SOP) [7]. We cannot exclude the fact that some participants acted according to their familiar SOP or local resistant patterns of bacteria in the natural work environment in contrast to national guidelines. However, a surprisingly low number of participants (19.4%) choose an antibiotic treatment with a proper dose adjustment to renal function and length of therapy, which only increased to 30.3% when the length of therapy was not considered in the analysis. In the only study analyzing the impact of a CDSS on compliance with guidelines on antibiotic treatment of urinary tract infection, around 30% of clinicians named the appropriate antibiotic and duration [18]. Our study points to the fact that medical doctors require support through information tools in their daily routine [22].

### CDSS and conventional information tools

Young medical doctors use online information regularly for treatment recommendations and diagnosis findings in their daily routine [23]. Traditional analog media such as teaching manuals or pocket guides are used less frequently compared to digital information tools in Germany by medical professionals [11]. CDSS can improve the quality of antibiotic prescription, but their effect seems to be moderate [24, 25]. Medical doctors with less working experience benefit the most from tools like a CDSS [26]. In our study, we developed an easy to use CDSS containing guideline-based definitions for urinary tract infection entities and corresponding treatment options. Compared to conventional tools, the CDSS improved diagnosis findings and treatment recommendations in our study significantly. Even the group free internet access was not equal to CDSS. However, often participants did not use the assigned information tool, mostly medical doctors compared to medical students (data not shown). Studies have shown that a significant obstacle in implementing a successful CDSS is medical doctors, as they often assume that they are experts and do not require support [20]. However, in the case of participants who used the assigned CDSS, 62.1% provided a correct diagnosis and 73.7% a correct treatment recommendation. In the study by Demonchy et al., the CDSS was only able to improve the rate of appropriate antibiotic and duration from 33% (no intervention) to 53% significantly in one out of three study centers [18]. The two other study centers did not show a difference, which raised questions if the CDSS is effective at all. Our study supports the fact that CDSS can improve adherence to guidelines in urinary tract infection as it is shown in other disease entities, too [8]. Although better than conventional tools, the CDSS results were less than perfect in our study, which could be due to its first-time use without prior introduction. Alternatively, participants did not base their decision on the CDSS, because they disagreed with the proposed results. Nevertheless, 73.8% of CDSS users based their decision on the CDSS recommendations, and they were more confident in their results than users of conventional tools.

The success of CDSS systems depends on its design and the organizational structure and environment in which it is implemented. Easy access and intuitive handling are essential factors for successful CDSS in the daily routine [25, 26]. In this regard, processing time in our study was similar between CDSS and free Internet access and other conventional tools, which argues for easy and intuitive handling of our system.

## Conclusions

The presented data suggest that medical personal do require additional information in finding the proper diagnosis hence treatment recommendation for urinary tract infection. Our study was done with a small number of participants, and the results are only representative for the analyzed population and not for a medical population treating daily this kind of infectious disease. The knowledge and correct application of current treatment guidelines would have been sufficient. However, the overestimation of one’s capabilities may prevent a rational antibiotic treatment recommendation in real life. Digital media such as CDSS are an excellent way to overcome this challenge if they provide easy access and intuitive handling in the daily routine, especially for young and inexperienced medical doctors. The efficacy of our or other CDSS need to be tested in real-life situations. However, one advantage of CDSS in contrast to web-based information like free internet access or analog media like pocket guides is that information can easily be adapted to the local situation in the form of a local SOP.

## Supplementary information

**Additional file 1: Figure S1.** Questionnaire Part 1.

**Additional file 2: Figure S2.** Questionnaire Part 2.

**Additional file 3: Figure S3.** CDSS “Infektionsnetzwerk/Antibiotix” – software developed for the study: A) A search field allows to enter the assumed diagnosis. B) user can enter disease characteristics or symptoms. C) the software suggests a list of potential diagnosis ordered by likelihood.

**Additional file 4: Figure S4.** Questionnaire Part 1 – case description.

**Additional file 5: Figure S5.** Distribution of medical doctors (dark grey) and medical students (light grey) in groups CDSS = Clinical Decision Support System, FIA = Free Internet Access, PPG = Pharmaceutical Pocket Guide, PGAT = Pocket Guide Antibiotic Therapy, control = no information tool (absolute numbers of participants per group per education level).

## Data Availability

Datasets used and analyzed during the current study are available from the corresponding author on reasonable request. All personal identifiers were removed from the dataset.

## Abbreviations

ABS: Antibiotic Stewardship
AU: As-used
CDSS: Clinical Decision Support System
CRP: C-reactive protein
DGIM: German Society for Internal Medicine
DGVS: German Society for Digestive and Metabolic Diseases
GFR: Glomerular filtration rate
ITU: Intention-to-use
m: Minute
MDRD-GFR: Modification of Diet in Renal Disease- glomerular filtration rate
s: Second
SOP: Standard operating procedure
