# Supplementary material for: A clinical decision support system improves antibiotic therapy for upper urinary tract infection in a randomized single-blinded study
Source: BMC Health Serv Res. 2020 Mar 6;20:185. doi: 10.1186/s12913-020-5045-6 (PMC7059328; doi:10.1186/s12913-020-5045-6)
Supplement: Supplementary file 1 — Additional file 1: Figure S1. Questionnaire Part 1. [file 12913_2020_5045_MOESM1_ESM.docx]

### Participant ID: ___ ___ ___ ___

### Questionnaire Part 1

*„Studie zur* **Ve**rbesserung der **r**ationalen Verschreibung von **A**ntibiotika“
(VerA-Studie)

This questionnaire is anonymized with an ID, which can’t be used to identify the person. Please answer the questions carefully.

**Age:** ________

**Gender:** ○ female ○ male

**Profession:** ○ doctor ○ medical student

***For medical doctors***

**Working experience as medical doctor: ___________ years**

Please summarize all your working experiences

**Where do you work currently?** ○ hospital ○ practitioner

**If you work in a hospital, what is your current position?**

○ Resident ○ senior doctor ○ head of department

**Are you a medical specialist?**

○ no ○ yes, I’m specialist for: ____________________________________

***For medical students***

**Medical school:**

________________________________________________________________________

**Current year of training:** ○ year 1-2 ○ year 3-5 ○ year 6
