# Supplementary material for: A clinical decision support system improves antibiotic therapy for upper urinary tract infection in a randomized single-blinded study
Source: BMC Health Serv Res. 2020 Mar 6;20:185. doi: 10.1186/s12913-020-5045-6 (PMC7059328; doi:10.1186/s12913-020-5045-6)
Supplement: Supplementary file 2 — Additional file 2: Figure S2. Questionnaire Part 2. [file 12913_2020_5045_MOESM2_ESM.docx]

### Participant ID: ___ ___ ___ ___

### Questionnaire Part 2

*„Studie zur* **Ve**rbesserung der **r**ationalen Verschreibung von **A**ntibiotika“
(VerA-Studie)

This questionnaire is anonymized with an ID, which can’t be used to identify the person. Please answer the questions carefully.

**How confident are you that your diagnosis and therapy are correct?**

| **very confident**  **1** | **confident**  **2** | **less confident**  **3** | **little confident**  **4** | **uncertain**  **5** | **very uncertain**  **6** | **don’t know** |
| --- | --- | --- | --- | --- | --- | --- |
| **○** | **○** | **○** | **○** | **○** | **○** | **○** |

**Did you use the assigned information tool?**

| - **No**, I didn’t use it - **Yes**, but my decision was not based on the information tool - **Yes**, my diagnosis/therapy was based on the information tool |
| --- |

**Do you think that using a different information tool would have improved your result?** (multiple choice)

- No
- Yes, using CDSS
- Yes, using free internet access
- Yes, using a pocket guide
- Yes, using: ____________________________________________________

**Did you consider one of the following?**

- Dose adjustment because of renal failure
- Interaction of medications
- Adjustment due to bacterial resistance
- Costs

***Only for participants using CDSS***

**Did you use the CDSS provided in this study before?**

○ yes ○ no

**Would you use the CDSS regularly in the daily routine?**

○ yes ○ no

**Do you think this CDSS could improve the quality of antibiotic prescription?**

○ yes ○ no

**Please rate the usability and efficiency of this CDSS**

| **very good**  **1** | **good**  **2** | **satisfactory**  **3** | **sufficient**  **4** | **poor**  **5** | **insufficient**  **6** | **don’t know** |
| --- | --- | --- | --- | --- | --- | --- |
| **○** | **○** | **○** | **○** | **○** | **○** | **○** |

Thank you very much for your participation. We are happy about your comments.

**Please don’t pass on any information to other persons, who participate in the study. In this way you help us to conduct the study in an unbiased way.**

We would be glad to see you at our website www.infektionsnetzwerk.de.

**Comments:**
