# Supplementary figures and images for: A clinical decision support system improves antibiotic therapy for upper urinary tract infection in a randomized single-blinded study
Source: BMC Health Serv Res. 2020 Mar 6;20:185. doi: 10.1186/s12913-020-5045-6 (PMC7059328; doi:10.1186/s12913-020-5045-6)

## Slide 1
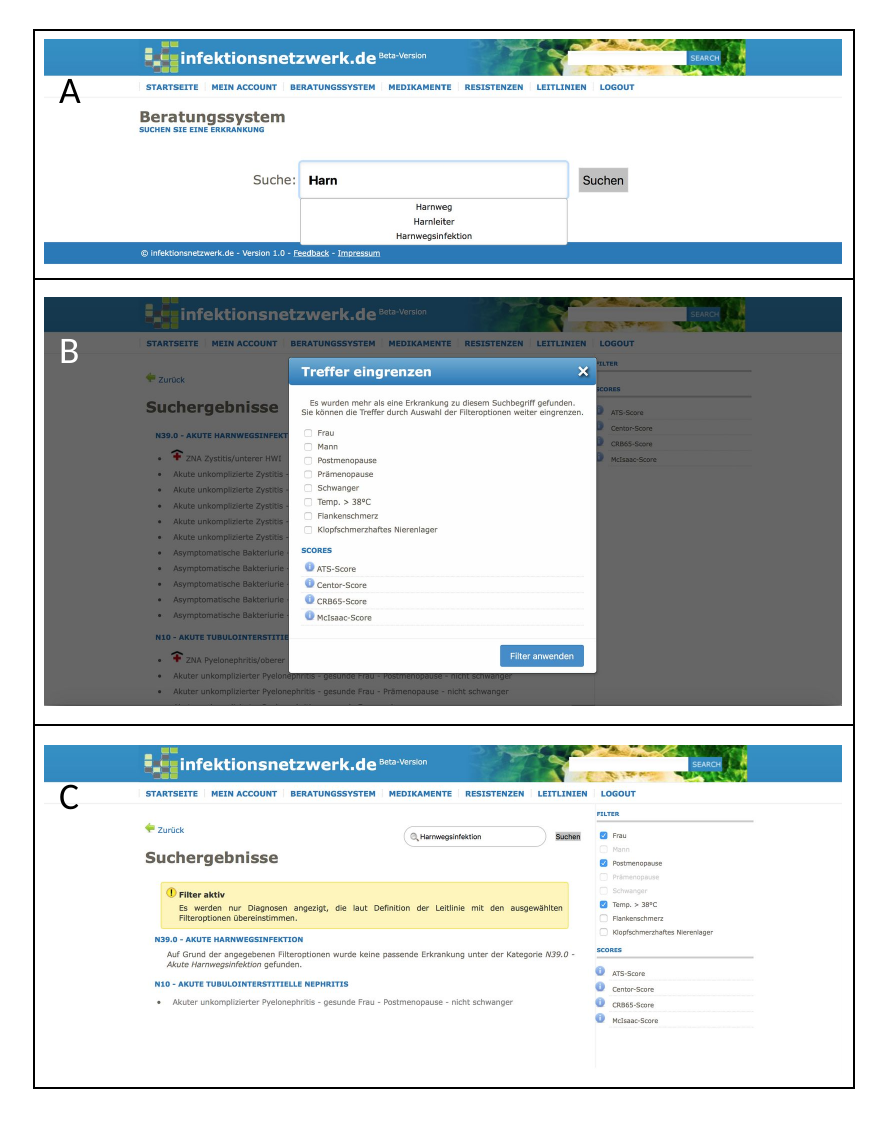

A
B
C

Supplement: Supplementary file 3 — Additional file 3: Figure S3. CDSS “Infektionsnetzwerk/Antibiotix” – software developed for the study: A) A search field allows to enter the assumed diagnosis. B) user can enter disease characteristics or symptoms. C) the software suggests a list of potential diagnosis ordered by likelihood. [file 12913_2020_5045_MOESM3_ESM.pptx]
