# Supplementary material for: A clinical decision support system improves antibiotic therapy for upper urinary tract infection in a randomized single-blinded study
Source: BMC Health Serv Res. 2020 Mar 6;20:185. doi: 10.1186/s12913-020-5045-6 (PMC7059328; doi:10.1186/s12913-020-5045-6)
Supplement: Supplementary file 4 — Additional file 4: Figure S4. Questionnaire Part 1 – case description. [file 12913_2020_5045_MOESM4_ESM.docx]

### Participant ID: ___ ___ ___ ___

### Questionnaire Part 1

*„Studie zur* **Ve**rbesserung der **r**ationalen Verschreibung von **A**ntibiotika“
(VerA-Studie)

A 64-year-old woman (80 kg; 168 cm) enters the emergency room (ER) because of fever (39°C) and burning while urinating. For the last 2 days, she had problems to empty her bladder and she used the bathroom about 8 times a day only with small urine portions, no vaginal discharge.

In the ER a urine dip stick is positive for leucocytes, nitrate and negative for glucose, no renal angle tenderness, body temperature 38.7°C.

Medical history: arterial hypertension, diabetes mellitus type 2. Medication: Ramipril 5mg q.d., Metformin 500mg b.i.d., Calcium/Vitamin D3 q.d..

Lab work: leucocytes 14.7*10^9^/l [4.2-10.2*10^9^/l], CRP 112 mg/l [0-5mg/l], creatinine 114,9 µmol/l [58,3-96,4 µmol/l], MDRD-GFR 41.2 ml/min/1.73m^2^, serum glucose level 7.94 mmol/l [4.1-6.1 mmol/l].

Blood and urine cultures pending.

**Please provide a diagnosis and recommend an empiric antibiotic therapy:**

**Diagnosis (**please be as specific as possible**): ______________________________________**

**__________________________________________________________**

**Antibiotic treatment** (active substance or trade name)

**Route of administration: ○ i.v. ○ p.o. ○ i.m.**

**_____________________________ _________ x daily for ________ days**

**Dose** (incl. unit)

**Additional comments / comments about therapy:**
