# Supplementary material for: A clinical decision support system improves antibiotic therapy for upper urinary tract infection in a randomized single-blinded study
Source: BMC Health Serv Res. 2020 Mar 6;20:185. doi: 10.1186/s12913-020-5045-6 (PMC7059328; doi:10.1186/s12913-020-5045-6)
Supplement: Supplementary file 5 — Additional file 5: Figure S5. Distribution of medical doctors (dark grey) and medical students (light grey) in groups CDSS = Clinical Decision Support System, FIA = Free Internet Access, PPG = Pharmaceutical Pocket Guide, PGAT = Pocket Guide Antibiotic Therapy, control = no information tool (absolute numbers of participants per group per education level). [file 12913_2020_5045_MOESM5_ESM.pptx]

## Slide 1
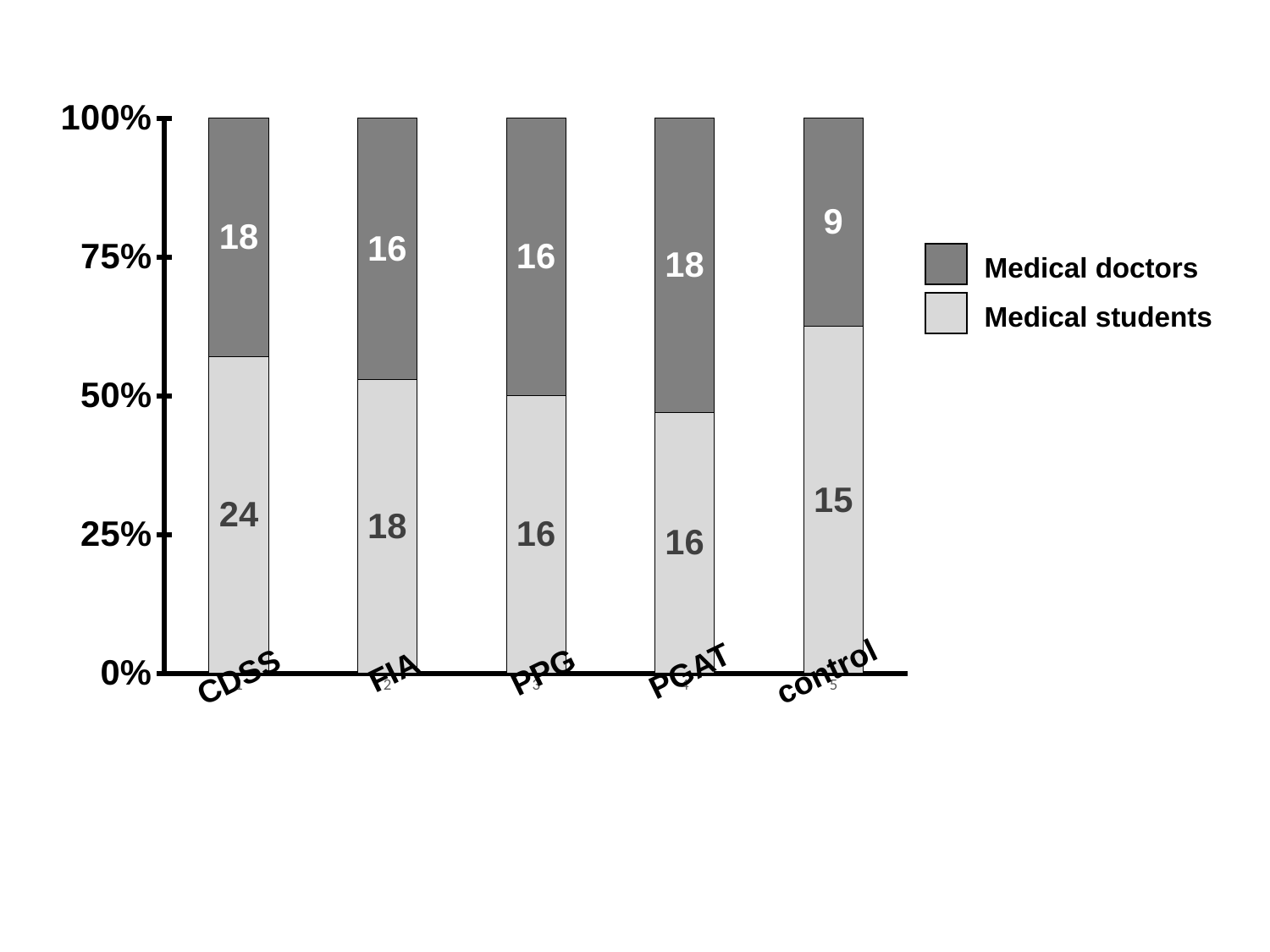

### Chart
| Category | Studierende | Ärzte |
|---|---|---|
Medical doctors
Medical students
FIA
PGAT
PPG
control
CDSS
